# Preparation and Characterization of a Dry Powder Inhaler Composed of PLGA Large Porous Particles Encapsulating Gentamicin Sulfate

**DOI:** 10.15171/apb.2019.029

**Published:** 2019-06-01

**Authors:** Farideh Shiehzadeh, Mohsen Tafaghodi, Majid-Laal Dehghani, Faezeh Mashhoori, Bibi Sedigheh Fazly Bazzaz, Mohsen Imenshahidi

**Affiliations:** ^1^School of Pharmacy, Mashhad University of Medical Sciences, Mashhad, Iran.; ^2^Student Research Committee, Mashhad University of Medical Sciences, Mashhad, Iran.; ^3^Nanotechnology Research Center, Pharmaceutical Technology Institute, Mashhad University of Medical Sciences, Mashhad, Iran.; ^4^Biotechnology Research Center, Pharmaceutical Technology Institute, Mashhad University of Medical Sciences, Mashhad, Iran.; ^5^Department of Pharmaceutical Control, School of Pharmacy, Mashhad University of Medical Sciences, Mashhad, Iran.; ^6^Department of Pharmacodynamics and Toxicology, School of Pharmacy, Mashhad University of Medical Sciences, Mashhad, Iran.

**Keywords:** Aminoglycosides, Drug delivery systems, Dry powder inhalers, Gentamicin sulfate, Large porous particles, Lung, PLGA

## Abstract

***Purpose:*** Direct delivery of aminoglycosides to the lungs was under extensive evaluations during the last decades. Because of large particle size, low density and porous structure, large porous particles (LPPs) are versatile carriers for this purpose. In this study, poly (lactic-co-glycolic acid) (PLGA) LPPs encapsulating gentamicin sulfate were prepared and *in vitro* characteristics of their freeze-dried powder as a dry powder inhaler (DPI) were evaluated.

***Methods:*** To prepare PLGA LPPs, a double emulsification-solvent evaporation method was optimized and gentamicin sulfate was post-loaded in the LPPs. *in vitro* characteristics including morphological features, thermal behavior, aerodynamic profile and cumulative drug release were evaluated by the scanning electron microscope (SEM), differential scanning calorimetry (DSC), next-generation cascade impactor (NGI) and Franz diffusion cell respectively.

***Results:*** The obtained results revealed that the preparation method was capable to produce spherical large homogenous highly porous particles. 94% of gentamicin sulfate released from LPPs up to 30 minutes. Mass median aerodynamic diameter (MMAD) and fine particle fraction (FPF) were 4.9 µm and 39% respectively.

***Conclusion:*** In this study, dry powder formulation composed of PLGA LPPs encapsulating gentamicin sulfate showed a promising *in vitro* behavior as a pulmonary delivery carrier. Improvements on the aerodynamic behavior and *in vivo* evaluations recommended for further developments.

## Introduction

Local delivery of antibiotics directly to the pulmonary infections extensively studied during the last decades. Because of the broad antibiotic spectrum, aminoglycosides were under the most evaluations for this purpose. Their high polarity has reduced their penetration ability through epithelia; therefore, high parenteral doses are needed to provide the therapeutic concentrations in the alveoli. These high doses may lead to some severe systemic side effects including renal impairment, neuromuscular toxicity and arising new bacterial resistance. Therefore, attempts have been focused on shifting their systemic administration to the local delivery.^1,2^

Gentamicin sulfate is a member of this antibiotic family and as it is effective against the *Pseudomonas aeruginosa*, it has been applied in the pulmonary infections including cystic fibrosis, bronchiectasis, etc. However, because of the hygroscopic nature of its raw material, its formulation and delivery process encountered several challenges.^3,4^

Vehicle mediated drug delivery is one of the solutions to overcome the unwanted physicochemical properties of the active pharmaceutical ingredients and optimize the aerodynamic characteristics for delivery via the pulmonary route.^5^ Porous polymeric particles are versatile particulate drug delivery systems in this regard. Their highly internal porous structure enables them to encapsulate a large drug payload. Low density and large geometric size which provide the macrophage scape are among their well-known characteristics.^5-7^

The objective of this study was preparing and evaluating a dry powder inhaler (DPI) composed of the poly (lactic-co-glycolic acid) (PLGA) large porous particles (LPPs) which encapsulate gentamicin sulfate. The preparation method of PLGA LPPs was optimized and gentamicin sulfate was then encapsulated by the post-loading method. After freeze-drying, the *in vitro* characterizations were performed on the powder.

## Materials and Methods

PLGA 50:50 0.18 dL/g (Resomer^®^ 502 H) was purchased from Boehringer Ingelheim, Ingelheim am rhein, Germany. Gentamicin sulfate was donated by Alborz Darou, Qazvin, Iran. Polyvinylalcohol, Dichloromethane, Ammonium bicarbonate orthophetaldialdehyde (OPA), mannitol, and sodium borate 10-hydrate were purchased from Merck, Darmstadt, Germany. All the other chemicals used were of analytical grade.

### Preparation of PLGA LPPs

#### Preparation of blank PLGA LPPs (Double emulsification-solvent evaporation method (W/O/W))

To prepare blank LPPs, primarily 2 mL of a 40 mg/mL PLGA in dichloromethane solution was prepared. Then 0.4 mL of a 1.5% freshly prepared ammonium bicarbonate solution was added, and the whole system was probe-sonicated (Soniprep 150, Fisons, Sussex, England) for 90 seconds (25°C, 25% amplitude). The secondary emulsion was prepared by homogenization of the primary emulsion in 25 mL of polyvinylalcohol 1% solution in 8000 rpm for 30 seconds. Subsequently, 30 mL of deionized water was added to the whole formulation and stirred (1000 rpm) overnight. Particles were finally washed for three times with deionized water and freeze-dried while contained 3% w/v mannitol as a cryoprotectant and to improve the aerodynamic behavior of the powder.^8,9^

### Drug loading process (post-loading)

A known amount of freeze-dried blank PLGA LPPs was dispersed in gentamicin sulfate (30% w/w) aqueous solution. After 5 minutes of constant shaking, the suspension was freeze-dried eventually. To analysis the drug encapsulation efficiency%, a known volume of suspension was taken. The particles then were collected by centrifugation at 4000 rpm for 5 min and lyophilized for further analysis (n=3).

### Characterization of PLGA LPPs

#### Production yield and morphology

The production yield of the PLGA LPPs was calculated as the ratio of the final mass of the freeze-dried powder to the primary mass of the raw materials.

Morphology of freeze-dried PLGA LPPs was studied by scanning electron microscopy (SEM) (Stereoscan-360 Cambridge, England). Prior to the microscopy, particles surface was coated with gold to improve the picture resolution by intensifying the electrons emission.^10^

#### Flowability of DPI

Hausner ratio and Carr’s compressibility index (CI) were calculated to evaluate the flow properties of the freeze-dried powder. These were determined from the tapped ρ_tap_ and bulk ρ_bulk_ density values using the following equations^10,11^:

CI = (1-ρ_bulk_/ρ_tap_)*100 Eq. (1)

*Hausner ratio* = 100/ (100-CI) Eq. (2)

To determine the bulk and tapped density of the freeze-dried drug-loaded PLGA LPPs, a known amount (m=180±2 mg) of the powder was loaded in a 5 mL guarded glass cylinder and its primary volume was recorded as *v*_0_. Then it was tapped (up to 14 mm of the ground) to a volume plateau (500 times). The secondary volume was recorded as *v*_1_. Bulk and tapped densities were calculated based on the following equations (n=3)^12^:

ρ_bulk_ = m/v_0_ Eq. (3)

ρ_tap_ = m/v_1_ Eq. (4)

#### Quantification of gentamicin sulfate

OPA was used as a derivatizing reagent and prepared as described previously.^13^ OPA interacts with the amino groups in the gentamicin molecule and converts them to chromophores with maximum absorption at 332 nm.^13^ Equal ratios of an unknown sample, OPA reagent, and isopropyl alcohol were mixed, protected from sunlight. A blank sample was also included. After 30 minutes, absorptions were recorded in 332 nm by Spectrophotometer (RF-540, Shimadzu, Tokyo, Japan). These methods were validated by a microbial bioassay on *Staphylococcus epidermidis* (data was not presented).

To determine the recovery of gentamicin sulfate from the freeze-dried drug-loaded PLGA LPPs, 10 ± 0.5 mg of the powder was suspended in 1 mL of dichloromethane by sonication for 5 min. To isolate gentamicin sulfate, the suspension was centrifuged in 10 000 rpm for 15 minutes. The precipitated plate was re-suspended in 1 mL of deionized water and concentration of gentamicin sulfate was calculated according to a linear standard plot (10-30 µg/mL). Recovery yield was calculated as the percentage of the recovered gentamicin sulfate from the final dry powder (n=3).^12,14,15^ The encapsulation efficiency was also determined as followed:

*EF% = mass of drug in LPPs/mass of drug used in formulation* Eq. (5)

#### Thermal analysis

Thermal behavior of the freeze-dried PLGA LPPs was evaluated by differential scanning calorimetry (DSC). 3 mg of drug-loaded PLGA LPPs, blank PLGA LPPs, physical mixture of blank PLGA LPPs and gentamicin sulfate powder, and gentamicin sulfate powder were placed in the aluminum panes and located in the thermal analyzer instrument (Star SW 7.0, Mettler Toledo, Greifensee, Switzerland). Under the nitrogen flow the temperature increased by the rate of 10°C/min up to 300°C and the endothermic/exothermic peaks were recorded.^16^

#### In vitro aerodynamic characterization

Aerodynamic characterization of the freeze-dried PLGA LPPs was performed by the next generation cascade impactor (NGI) (Copley, Nottingham, UK). 60 L\min airflow provided by a high capacity pump (HCP5, Copley, UK) and measured by a flowmeter (DFM2, Copley Scientific, UK). Applying a self-modified Spinhaler^®^ device, 50 ±2 mg of drug-loaded PLGA LPPs powder was aerosolized. Each test was done for 4 s to provide 4-L air flowing across the device. After each run, the retained powder on each stage was dissolved in 2 mL of acetone. As gentamicin sulfate and mannitol were not soluble in acetone, the suspension was centrifuged in 10 000 rpm for 15 minutes and the precipitant was dissolved in 1 ml of deionized water. The concentration of gentamicin sulfate was analyzed as described earlier. Using CITDAS software (V. 3.0), the aerodynamic characteristics including mass median aerodynamic diameter (MMAD), emitted dose and fine particle fraction (FPF) were calculated.^17,18^ All the experiments were performed in triplicate.

#### In vitro release

The release profile of the drug-loaded PLGA LPPs was studied by Franz diffusion cells. Each cell was filled with 25 mL of phosphate buffer saline, as a receptor chamber (pH 7.4). A 0.45-µm membrane filter was used as a barrier between two compartments. A known amount of the freeze-dried drug-loaded PLGA LPPs was placed on the membrane as the donor compartment. One cell considered as the negative control and treated by the blank PLGA LPPs. The whole system was thermostated by a 37°C water jacket. Sampling was done every 10 minutes for 30 minutes and thereafter every 30 minutes up to 240 minutes. After each sampling, the aspirated buffer was replaced by the fresh buffer. Gentamicin sulfate concentration was analyzed at each time point, and the cumulative release profile was determined (n=3).^16^

#### Stability assessment

Freeze-dried ‌drug-loaded PLGA LPPs were preserved in the glass container at room temperature protected from sunlight. After 6 months the particles were re-suspended in deionized water. The particle size and morphology were assessed using the optical microscope. Drug content of the powder was also determined as previously described.

#### Statistical analysis

Data were statistically evaluated by one-way analysis of variance (ANOVA) followed by Tukey-Kramer as the post-test, using GraphPad InStat 3.0. A *P* value equal to or less than 0.05 was considered as statistically significant. Data were presented as mean ± standard deviation (SD).

## Results and Discussion

### Preparation and drug loading of PLGA LPPs

The pioneer studies on the LPPs date back to the late 1990s when Edward and his colleagues introduced these polymeric particles as suitable pulmonary delivery carriers that can escape from macrophages uptake. Therefore, they extend the presence of active ingredients in the alveoli.^6^ Since then, different researches were conducted in this field. Proofs of that, through a preliminary Medline search, about one hundred papers were found. Among different cargoes that had been encapsulated in these carriers, aminoglycoside antibiotics were also found. In a study conducted by Garcia-Contreras et al, Capreomycin PLGA LPPs which were produced by spray drying were effective to reduce the microbial load of the TB infected lung of the animals in comparison with the other treatment groups.^19^ In another study by Giovagnoli et al, capreomycin loaded PLGA LPPs with desirable characteristics was prepared by the W/O/W method.^14^

To our knowledge, this is the first study on the PLGA LPPs encapsulating gentamicin sulfate. At the preliminary experiments of this study, we focused on optimizing the PLGA LPPs preparation method (data is not presented). Primary and secondary emulsifications were performed in different homogenization/sonication conditions; also, by the other mixer devices like magnetic and paddle stirrers.^14,15,20,21^ Particle’s surface morphology of all the prepared batches was evaluated using the optical microscopy. Production yields were also calculated. Based on the production yield, size, morphology and porosity, the ideal preparation method was chosen. Applying a porogen agent in the process has been one of the main strategies to prepare the LPPs. Different porogen agents were applied in the literature such as Pluronic F127,^22^ H2O2,^15^ methylene chloride,^23^ cyclodextrin,^24^ and ammonium bicarbonate. Because of biocompatibility and safety considerations in this study, ammonium bicarbonate was selected.^7^ It was also proved by increasing the internal aqueous phase volume of the primary emulsion, highly porous PLGA LPPs with significantly larger sizes were produced. However, their MMAD and aerodynamic behavior were not suitable for pulmonary delivery (Data was not included). Because of high water solubility of gentamicin sulfate and its small molecule and in order to eliminate the loss of drug during the washing process, it was post-loaded in the PLGA LPPs.^7,12,14,15,25^

### Characterization of Freeze-dried PLGA LPPs

#### Preparation yield

Among the different preparation methods, W/O/W emulsification method described in “material and methods” section provided the lowest polymer loss and the particles were formed by a high production yield (72±3.5%).

### Particles morphology

**Figure 1:**
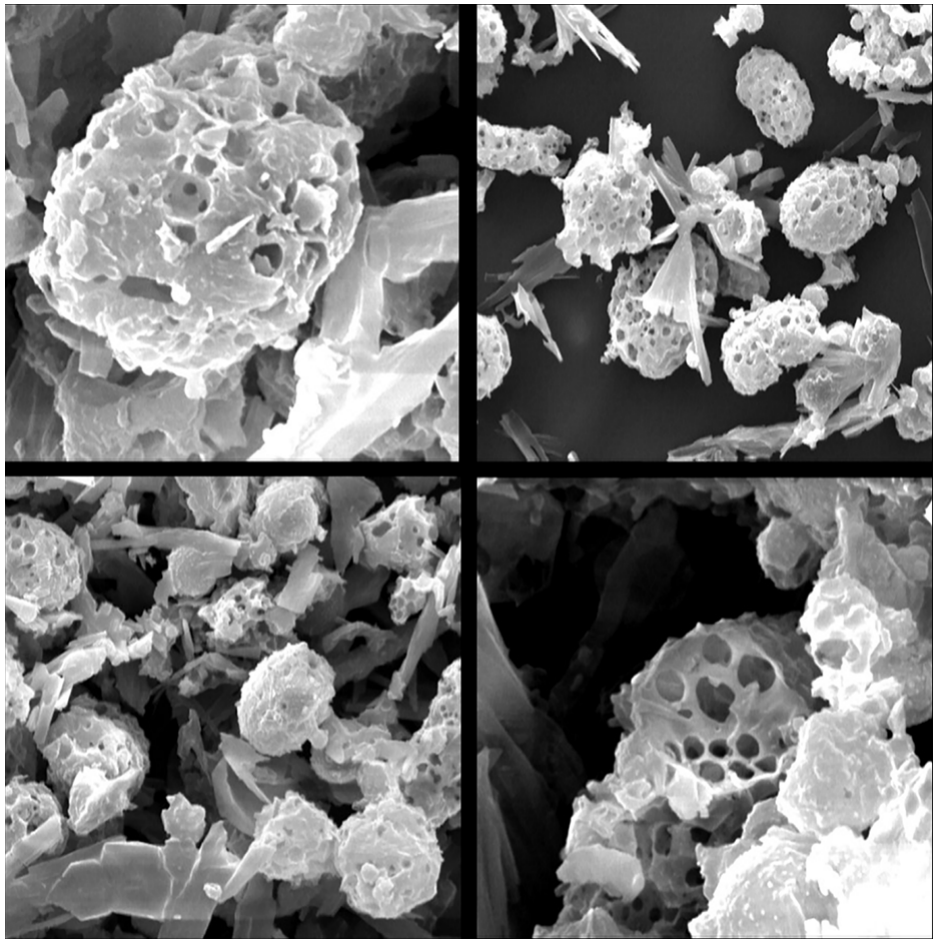
The scanning electron microscope (SEM) images of freeze-dried poly (lactic-co-glycolic acid) (PLGA) large porous particles (LPPs).

SEM was performed to study the morphology and structure of freeze-dried PLGA LPPs. As it was shown in Figure 1, the spherical homogenous highly porous particles with a geometric particle size of higher than 10 µm were formed. Van der Waals forces and consequently the aggregation tendency are at the minimum level when the particles are spherical and homogenous. This indicated suitable aerosolization of the PLGA LPPs dry powder.^26^

### Flowability of DPI

The bulk and tapped densities of freeze-dried PLGA LPPs were 0.075±0.005 and 0.104±0.002 g/cm^3^ respectively. Therefore, CI and Hausner ratio were calculated as 28.17±3.57 and 1.39±0.069 respectively. The low density of the LPPs brought their aerodynamic diameter in the suitable range for pulmonary delivery (1-5 µm).^15^ Carr’s index values of <25 indicate a good flowability and values higher than 40 related to poor flow characteristics. The lower value of Hausner ratio (but it should be >2.0) also indicate the more desirable flow ability. Based on this, the freeze-dried PLGA LPPs showed acceptable flow properties.^27^

### Gentamicin sulfate quantification

In this study, a simple and precise method for quantification of gentamicin sulfate was applied. Different analytical methods were introduced in the literature including; enzyme immunoassay, polarization fluoroimmunoassay, capillary electrophoresis, chemiluminescence, and chromatographic assays.^28,29^ The microbial assay is one of the accepted common methods described in the reference literature for most of the antibiotics. While it is a simple and economic assay, it is time-consuming.^28^ Therefore in this study, the microbial analysis was solely used to validate the spectrophotometry assays. Microbial bioassay of gentamicin sulfate showed an accuracy of 96.15±0.08%. The precision of analysis for OPA spectrophotometry was ≥96%. Furthermore, by the OPA spectrophotometry method, 96.4±2% of the drug content was successfully recovered from the freeze-dried PLGA LPPs while the EE% of the drug in the PLGA LPPs was 93.2±3%

### Thermal analysis

**Figure 2:**
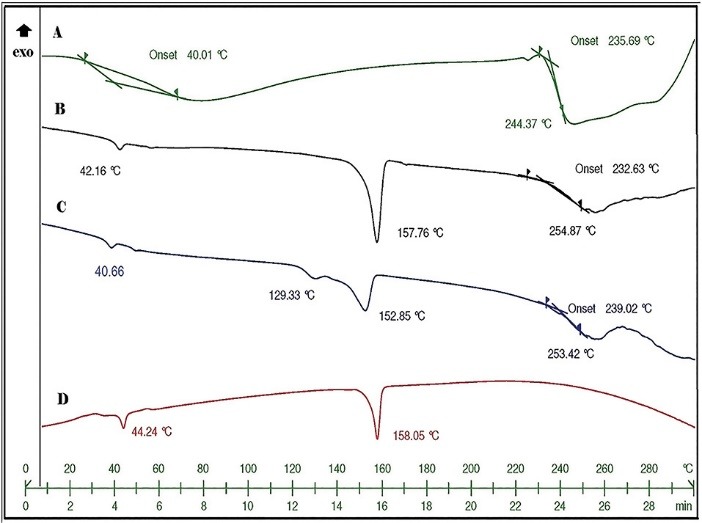
Differential scanning calorimetry (DSC) thermograms of A: Gentamicin sulfate, B: The physical mixture of gentamicin sulfate and Blank poly (lactic-co-glycolic acid) (PLGA) large porous particles (LPPs), C: Gentamicin sulfate loaded PLGA LPPs and D: Blank LPPs evaluated in 0-300 °C.

In the DSC diagram of gentamicin sulfate (Figure 2), two thermal events in 235.69°C and 244.37°C were recorded. These were related to the melting followed by degradation of the material. There was also a wide endothermic peak in 40°C which was related to loss of humidity. Hence, it was not found in the freeze-dried samples. In the samples that contain PLGA (B, C, and D), the endothermic event related to the glass transition of the polymer (40-44°C) was obvious. An endothermic event in 157.76-158.02°C which was related to the mannitol melting point was detected in the freeze-dried formulations (B, C, and D). This peak shifted to 152.58 °C in the drug encapsulated formulation.^15^ The thermal events of gentamicin sulfate in C were recorded in 254.87°C and 232.63°C. That could be related to the encapsulated gentamicin sulfate in the crystalline form or un-encapsulated part of the drug as no washing step was included after drug loading.

### In vitro drug release profile

**Figure 3:**
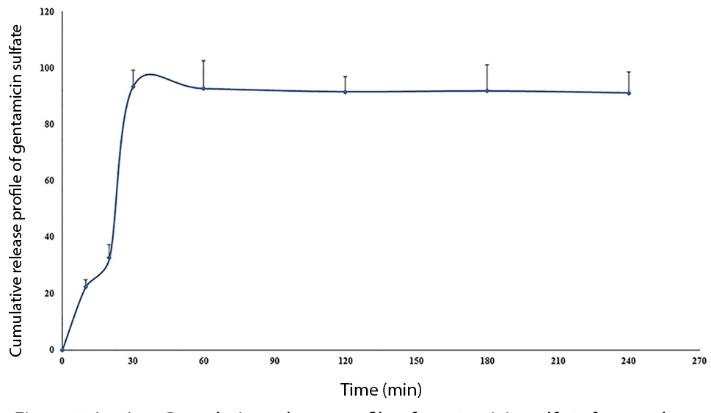
Cumulative release profile of gentamicin sulfate from poly (lactic-co-glycolic acid) (PLGA) large porous particles (LPPs) up to 240 minutes, measured by Franz diffusion cells in 37 °C (n=3).

The Franz diffusion cells are currently applied as the most resembling *in vitro* models to evaluate pulmonary dry powders release.^16^ The *in vitro* cumulative percent of released gentamicin sulfate from PLGA LPPs was presented in Figure 3.

From the plot, it could be simply distinguished that the total encapsulated drug was burst released up to 30 minutes (93.64±5.8%), and the curve reached the plateau after this time. The highly porous structure of the LPPs provided this fast and approximately complete drug release. Therefore, this formulation could be considered as a suitable drug delivery vehicle when the fast release of cargo is desirable.

### In vitro aerodynamic characterization

**Figure 4:**
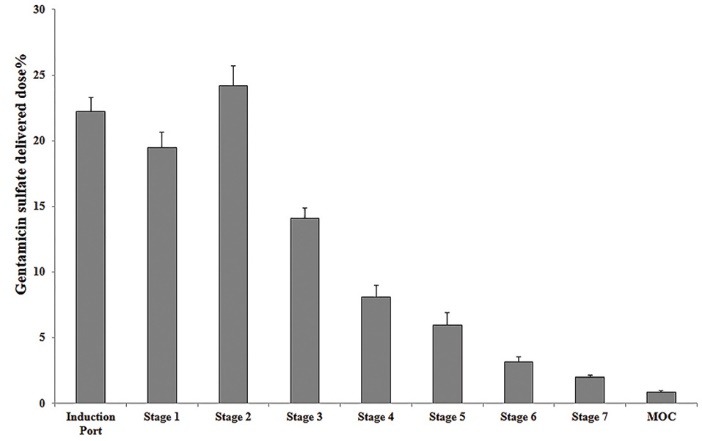
Dissemination percentage of the freeze-dried drug loaded poly (lactic-co-glycolic acid) (PLGA) large porous particles (LPPs) on next-generation cascade impactor (NGI) stages and induction port (n=3).

The aerodynamic characteristics of drug-loaded PLGA LPPs which were evaluated using the NGI were presented in Figure 4 and Table 1.

**Table 1:** Aerodynamic characteristics of the freeze-dried drug loaded poly (lactic-co-glycolic acid) PLGA large porous particles (LPPs) evaluated by next-generation cascade impactor (‌‌‌NGI)

	**Flow Rate (L/min)**	**R** ^2*^	**GSD**	**MMAD (µm)**	**Emitted dose (of 14 mg loaded dose)**	**FPF (%)**
**Freeze dried drug loaded PLGA LPPs**	60	0.99	2.92±0.7	4.98±0.1	12.6 mg	39.07±1.5

*GSD: geometric standard deviation, MMAD: mass median aerodynamic diameter, FPF: fine particle fraction, R2: coefficient of determination. Data were analyzed by CITDAS software (V. 3.0) (n=3).

Based on the obtained data, the MMAD and Geometric standard deviation (GSD, reflecting the normality of particle size distribution) were in the ideal range for the pulmonary delivery (1-5 and 1-3 µm respectively).^1,30^ The loaded dose in the device was emitted by the extent of 90% in each shot. FPF% was about 40% regarding the rest of ideal characteristics it considered an acceptable value.

### Stability assessment

**Figure 5:**
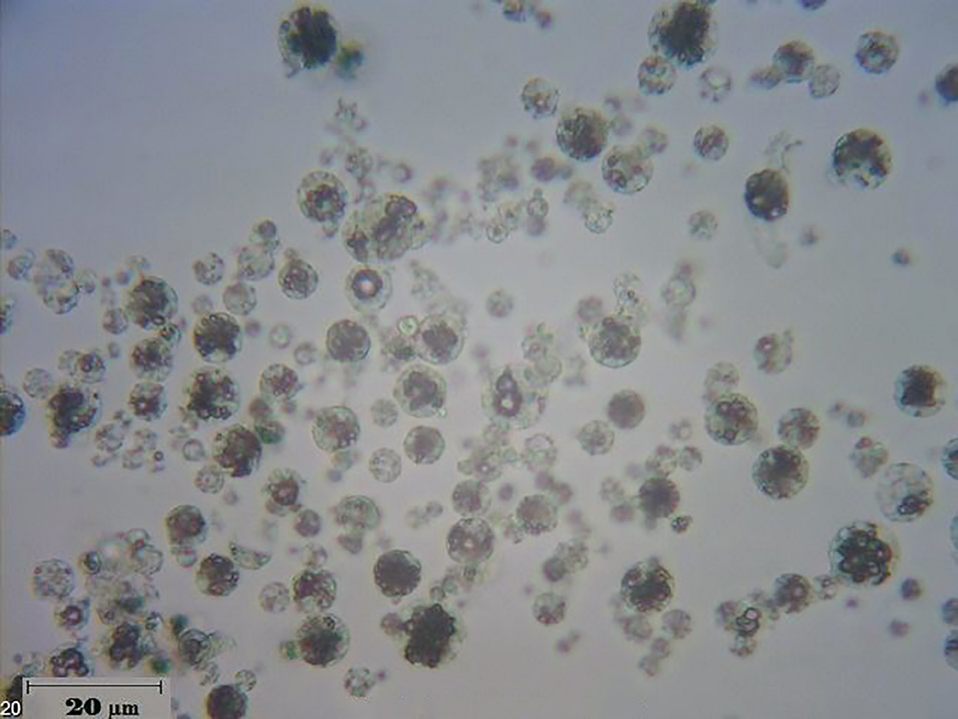
Optical microscope image of re-suspended drug loaded poly (lactic-co-glycolic acid) (PLGA) large porous particles (LPPs) in water after 6 months of preparation.

Based on the optical microscope image (Figure 5), the particle size and morphology were preserved and no aggregation was occurred. The drug content was also recovered by the extent of 97±0.5%.

## Conclusion

In this study, a simple and scalable method was optimized to prepare gentamicin sulfate PLGA LPPs powder suitable for pulmonary delivery. *In vitro* characterizations of the freeze-dried powder indicated that this formulation could be considered as a promising candidate for developments of local pulmonary antibiotherapy. However, some further modifications in the powder preparation method such as using pulmonary safe excipients to improve the aerodynamic behavior, involving un-encapsulated gentamicin sulfate in the release test to have a comparison, evaluating the cytotoxicity of the powder on the alveolar epithelial cells and *in vivo* evaluations would also shed the light on this formulation in the future.

## Ethical Issues

Not applicable.

## Conflict of Interest

There is no conflict of interests.

## Acknowledgments

This research was supported by Vice Chancellor for Research, Mashhad University of Medical Sciences, Mashhad, Iran.
